# Supplementary material for: Novel methods for epistasis detection in genome-wide association studies
Source: PLoS One. 2020 Nov 30;15(11):e0242927. doi: 10.1371/journal.pone.0242927 (PMC7703915; doi:10.1371/journal.pone.0242927)
Supplement: S1 File — (PDF) [file pone.0242927.s001.pdf]

S1 File. Genotypic hidden Markov model

In this Appendix, we explicit the transition and emission probabilities for the genotypic hidden Markov model. For that purpose, we start by considering a pair of ordered haplotypes $H^a = \left(H_1^a, \cdots, H_p^a\right) \in \{0,1\}^p$ and $H^b = \left(H_1^b, \cdots, H_p^b\right) \in \{0,1\}^p$. We recall that the two haplotypes correspond to the same positions. The hidden variables $Z^a = \left(Z_1^a, \cdots, Z_p^a,\right)$ and $Z^b = \left(Z_1^b, \cdots, Z_p^b,\right)$ represent cluster memberships. They take discrete values in $\{1, \cdots, K\}^p$. Scheet and Stephens [1] define the clusters as a "(common) combination of alleles at tightly linked SNPs". The underlying hidden Markov models for the two alleles have identical forms. We then focus on the first allele a. We follow the notations of [2].

The marginal distribution of the first hidden state can be written as:

$$q_1^{hap}(k) = \alpha_{1,k}, \quad k \in \{1, \cdots, K\}.$$

For $j \in \{2, \cdots, p\}$, the transition matrix Q_j^{hap} is given by:

$$Q_j^{hap}(k'|k) = P(H_j = k'|H_{j-1} = k') = \begin{cases} e^{-r_j} + \left(1 - e^{-r_j}\right)\alpha_{j,k'}, & k' = k \\ \left(1 - e^{-r_j}\right)\alpha_{j,k'}, & k' \neq k \end{cases}.$$

The parameter $r = (r_2, \cdots, r_p)$ can be assimilated to the recombination rate between loci $j-1$ and j, although Scheet and Stephens [1] point out the general mismatch between the observed recombination rates and the estimate of r. The parameter $\alpha = (\alpha_{j,k})_{(j,k)\in\{1,\cdots p\}\times\{1,\cdots,K\}}$ is the relative frequency of the cluster k in locus j.

Conditionally on the latent state $Z_j^{hap} = z_j$, the allele H_j is a Bernoulli random variable, $H_j|Z_j \sim \mathcal{B}(\theta_{j,z_j})$. θ_{j,z_j} is the frequency of allele 1 in cluster z_j at the position j:

$$f_j^{hap} = (h_j; z_j, \theta) = \begin{cases} 1 - \theta_{j,z_j}, & h_j = 0 \\ \theta_{j,z_j}, & h_j = 1 \end{cases}.$$

Under the Hardy-Weinberg equilibrium (HWE), a third hidden Markov model for the unphased genotype can be derived by combining the HMMs of the two alleles a and b. The emission states $X = (X_1, \cdots, X_p) \in \{0,1,2\}^p$ are given by the sum of the emission states, $H^a + H^b = \left(H_1^a + H_1^b, \cdots, H_p^a + H_p^b\right)$. Because of the phase indetermination, the latent states are unordered pairs of haplotype latent states, $Z = (\{Z_1^a, Z_1^b\}, \cdots, \{Z_p^a, Z_p^b\})$. Thus, the dimensionality of the latent variable space is $K(K+1)/2$. The different probabilities of the genotype model are computed by considering the two cases: $Z_j^a = Z_j^b$ and $Z_j^a \neq Z_j^b$.

The initial latent state distribution is given by:

$$q_1^{gen}(\{k^a, k^b\}) = \begin{cases} (\alpha_{1,k^a})^2, & k^a = k^b \\ 2\alpha_{1,k^a}\alpha_{1,k^b} & k^a \neq k^b \end{cases}.$$

In a similar fashion, the transition probabilities:

$$Q_j^{gen}(\{\underline{k}^a, \underline{k}^b\}|\{k^a, k^b\}) = \begin{cases} Q_j^{hap}(\underline{k}^a|k^a)Q_j^{hap}(\underline{k}^b|k^b) + Q_j^{hap}(\underline{k}^b|k^a)Q_j^{hap}(\underline{k}^a|k^b), & \underline{k}^a \neq \underline{k}^b \\ Q_j^{hap}(\underline{k}^a|k^a)Q_j^{hap}(\underline{k}^b|k^b), & \text{otherwise} \end{cases}.$$

and, the emission probabilities are

$$f_j(x_j; \{k^a, k^b\}, \theta) = \begin{cases} (1-\theta_{j,k^a})(1-\theta_{j,k^b}), & x_j = 0 \\ \theta_{j,k^a}(1-\theta_{j,k^b}) + (1-\theta_{j,k^a})\theta_{j,k^b}, & x_j = 1 \\ \theta_{j,k^a}\theta_{j,k^b}, & x_j = 2 \end{cases}.$$

For the estimate of the parameters $\nu = (\alpha, r, \theta)$, we use the imputation software fastPHASE [1] which fits the hidden Markov model using an expectation-maximization (EM) algorithm [3]. Its computational complexity is $\mathcal{O}(npK^2)$. The complexity scales linearly for both p and n, rendering fastPHASE well-suited for real case-control datasets where the number of SNPs is typically in the hundreds of thousands and the number of samples in the thousands. In practice, as a trade-off between a rich representation of the clusters and the ensuing quadratic complexity, we chose $K = 12$.

References

1. Scheet P, Stephens M. A fast and flexible statistical model for large-scale population genotype data: applications to inferring missing genotypes and haplotypic phase. American journal of human genetics. 2006;78(4):629–44. doi:10.1086/502802.

2. Sesia M, Sabatti C, Candès EJ. Gene hunting with hidden Markov model knockoffs. Biometrika. 2018;doi:10.1093/biomet/asy033.

3. Dempster AP, Laird NM, Rubin DB. Maximum Likelihood from Incomplete Data via the EM Algorithm. Journal of the Royal Statistical Society Series B (Methodological). 1977;39(1):1–38.
