# Supplementary material for: Novel methods for epistasis detection in genome-wide association studies
Source: PLoS One. 2020 Nov 30;15(11):e0242927. doi: 10.1371/journal.pone.0242927 (PMC7703915; doi:10.1371/journal.pone.0242927)
Supplement: S2 File — (PDF) [file pone.0242927.s002.pdf]

S2 File. Simulation results

1 First scenario: synergistic only effects

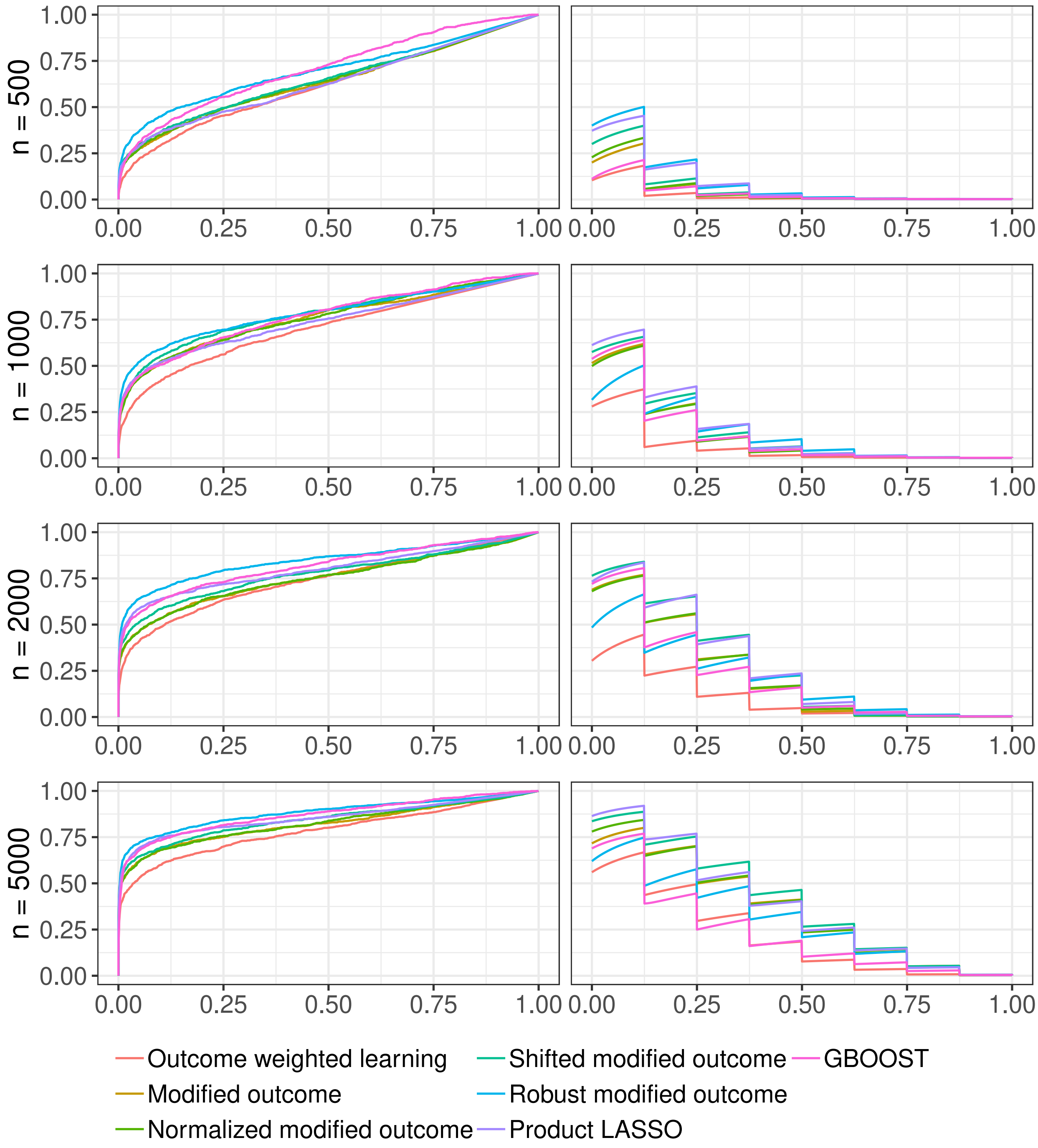

Fig A. Average ROC (left column) and PR (right column) curves for the first scenario

Table A. Average ROC and PR AUCs for the first scenario

Method	PR	ROC
n =500		
GBOOST	0.0362	0.7075
Modified outcome	0.0468	0.6747
Robust modified outcome	0.0973	0.7414
Normalized modified outcome	0.0512	0.6754
Shifted modified outcome	0.0644	0.6794
Outcome weighted learning	0.0254	0.6282
Product LASSO	0.0895	0.6514
n =1000		
GBOOST	0.1270	0.7688
Modified outcome	0.1284	0.7131
Robust modified outcome	0.1302	0.7434
Normalized modified outcome	0.1255	0.7120
Shifted modified outcome	0.1470	0.7224
Outcome weighted learning	0.0613	0.6764
Product LASSO	0.1619	0.7032
n =2000		
GBOOST	0.2103	0.8169
Modified outcome	0.2252	0.7512
Robust modified outcome	0.2070	0.8449
Normalized modified outcome	0.2266	0.7501
Shifted modified outcome	0.2704	0.7753
Outcome weighted learning	0.1045	0.7394
Product LASSO	0.2711	0.7989
n =5000		
GBOOST	0.2276	0.8697
Modified outcome	0.3512	0.8218
Robust modified outcome	0.3011	0.8818
Normalized modified outcome	0.3548	0.8248
Shifted modified outcome	0.3907	0.8423
Outcome weighted learning	0.2139	0.7847
Product LASSO	0.3779	0.8546

2 Second scenario: partial overlap between synergistic and marginal effects

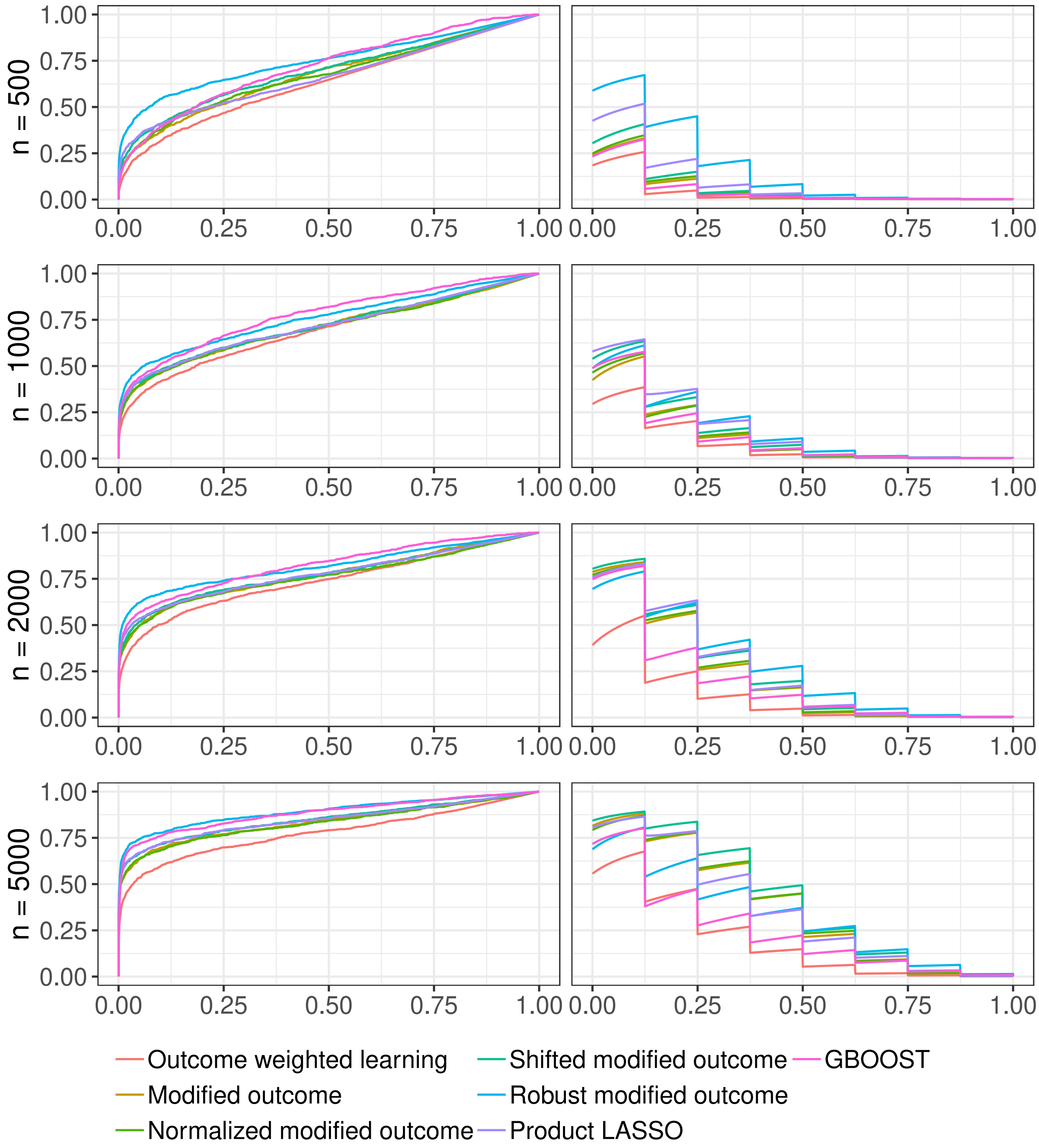

Fig B. Average ROC (left column) and PR (right column) curves for the second scenario

Table B. Average ROC and PR AUCs for the second scenario

Method	PR	ROC
n =500		
GBOOST	0.0516	0.7186
Modified outcome	0.0563	0.6750
Robust modified outcome	0.1716	0.7502
Normalized modified outcome	0.0590	0.6713
Shifted modified outcome	0.0712	0.6918
Outcome weighted learning	0.0367	0.6345
Product LASSO	0.0994	0.6659
n =1000		
GBOOST	0.1190	0.7773
Modified outcome	0.1195	0.7092
Robust modified outcome	0.1574	0.7601
Normalized modified outcome	0.1233	0.7080
Shifted modified outcome	0.1443	0.7160
Outcome weighted learning	0.0805	0.6923
Product LASSO	0.1609	0.7170
n =2000		
GBOOST	0.1933	0.8226
Modified outcome	0.2294	0.7708
Robust modified outcome	0.2732	0.8183
Normalized modified outcome	0.2321	0.7623
Shifted modified outcome	0.2532	0.7753
Outcome weighted learning	0.1114	0.7360
Product LASSO	0.2507	0.7762
n =5000		
GBOOST	02454	0.8821
Modified outcome	0.3718	0.8344
Robust modified outcome	0.3286	0.8916
Normalized modified outcome	0.3739	0.8309
Shifted modified outcome	0.4079	0.8487
Outcome weighted learning	0.1930	0.7769
Product LASSO	0.3537	0.8467

3 Third scenario: partial overlap between synergistic and quadratic effects

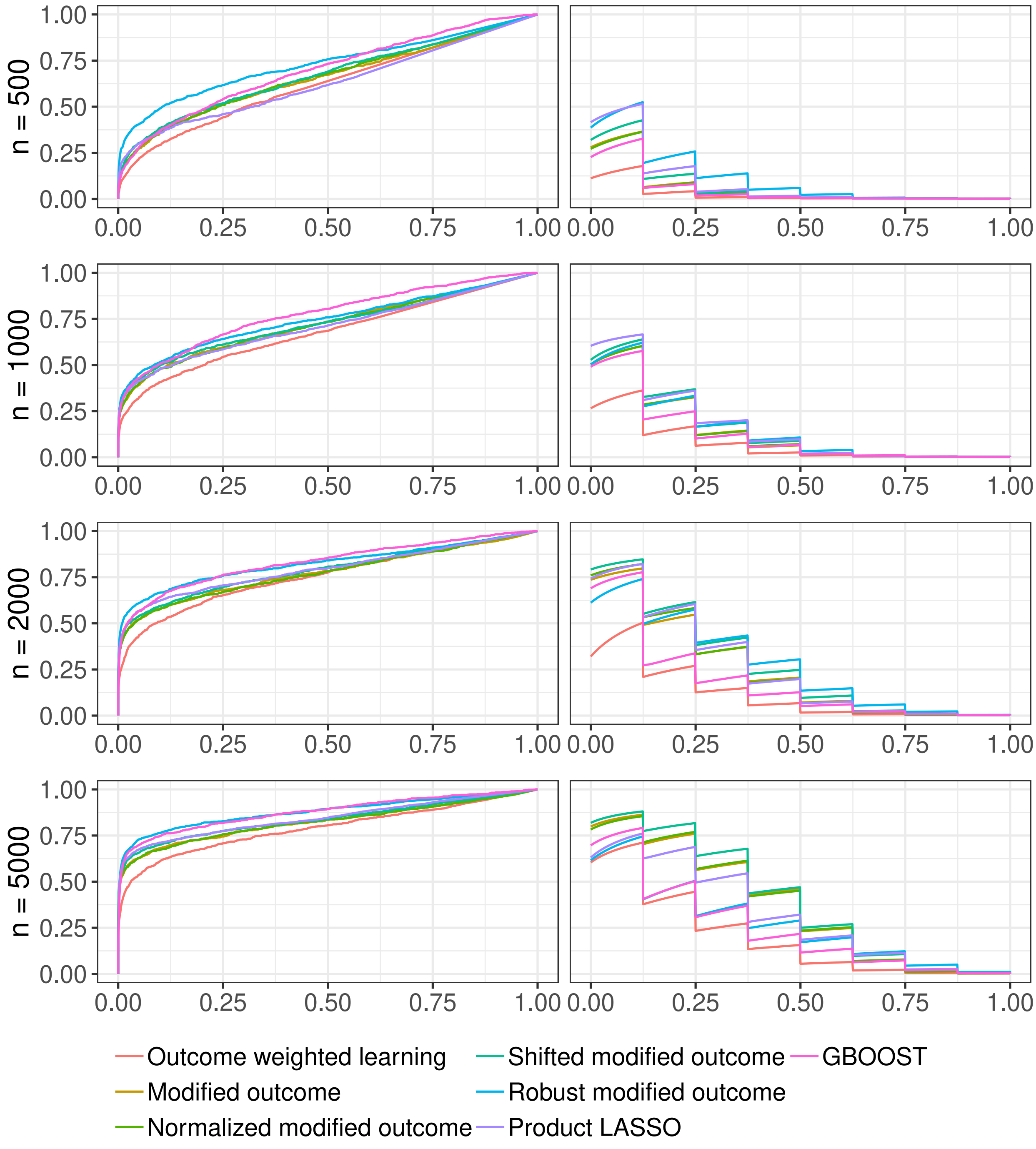

Fig C. Average ROC (left column) and PR (right column) curves for the third scenario

Table C. Average ROC and PR AUCs for the third scenario

Method	PR	ROC
n =500		
GBOOST	0.050	0.6970
Modified outcome	0.0570	0.6559
Robust modified outcome	0.1148	0.7296
Normalized modified outcome	0.0569	0.6627
Shifted modified outcome	0.0714	0.6703
Outcome weighted learning	0.0260	0.6233
Product LASSO	0.0889	0.6282
n =1000		
GBOOST	0.1228	0.7746
Modified outcome	0.1362	0.7181
Robust modified outcome	0.1513	0.7444
Normalized modified outcome	0.1373	0.7175
Shifted modified outcome	0.1546	0.7226
Outcome weighted learning	0.0728	0.6778
Product LASSO	0.1620	0.7100
n =2000		
GBOOST	0.1814	0.8307
Modified outcome	0.2430	0.7733
Robust modified outcome	0.2697	0.8235
Normalized modified outcome	0.2496	0.7724
Shifted modified outcome	0.2737	0.7886
Outcome weighted learning	0.1129	0.7535
Product LASSO	0.2543	0.7921
n =5000		
GBOOST	0.2467	0.8767
Modified outcome	0.3663	0.8241
Robust modified outcome	0.2660	0.8790
Normalized modified outcome	0.3669	0.8236
Shifted modified outcome	0.3944	0.8376
Outcome weighted learning	0.1965	0.7893
Product LASSO	0.3158	0.8439

4 Fourth scenario: partial overlap between synergistic and quadratic/marginal effects

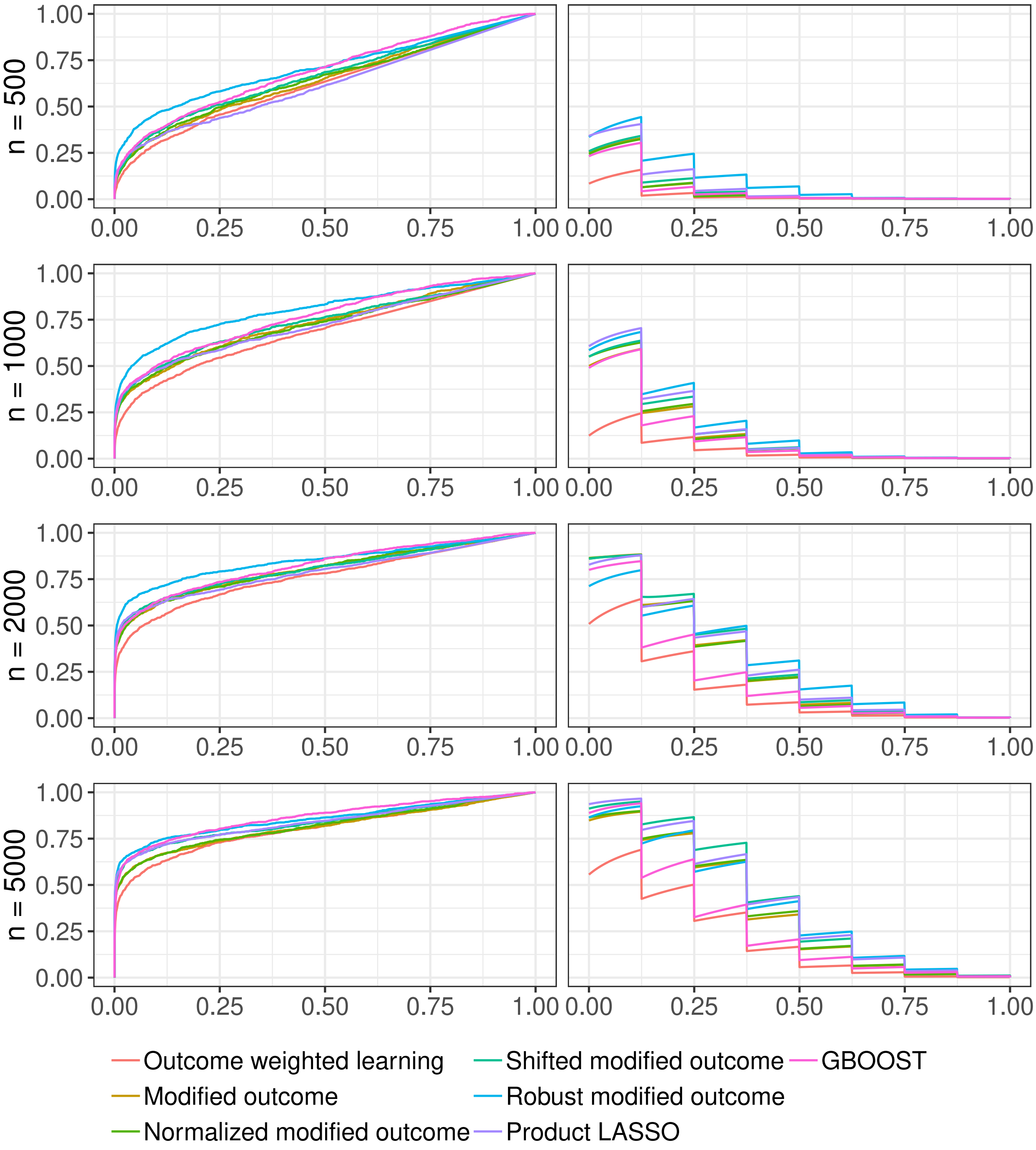

Fig D. Average ROC (left column) and PR (right column) curves for the fourth scenario

Table D. Average ROC and PR AUCs for the fourth scenario

Method	PR	ROC
n =500		
GBOOST	0.0479	0.6900
Modified outcome	0.0521	0.6427
Robust modified outcome	0.1066	0.7065
Normalized modified outcome	0.0513	0.6460
Shifted modified outcome	0.0591	0.6623
Outcome weighted learning	0.0227	0.6218
Product LASSO	0.0762	0.6174
n =1000		
GBOOST	0.1163	0.7647
Modified outcome	0.1283	0.7288
Robust modified outcome	0.1687	0.8049
Normalized modified outcome	0.1338	0.7200
Shifted modified outcome	0.1438	0.7388
Outcome weighted learning	0.0479	0.6838
Product LASSO	0.1554	0.7206
n =2000		
GBOOST	0.2129	0.8237
Modified outcome	0.2794	0.8007
Robust modified outcome	0.2986	0.8478
Normalized modified outcome	0.2763	0.8032
Shifted modified outcome	0.2960	0.8050
Outcome weighted learning	0.1530	0.7641
Product LASSO	0.2927	0.7899
n =5000		
GBOOST	0.2823	0.8656
Modified outcome	0.3541	0.8127
Robust modified outcome	0.3823	0.8568
Normalized modified outcome	0.3597	0.8175
Shifted modified outcome	0.4091	0.8388
Outcome weighted learning	0.2106	0.8031
Product LASSO	0.4000	0.8399
