# Supplementary material for: Novel methods for epistasis detection in genome-wide association studies
Source: PLoS One. 2020 Nov 30;15(11):e0242927. doi: 10.1371/journal.pone.0242927 (PMC7703915; doi:10.1371/journal.pone.0242927)
Supplement: Attachment — Submitted filename: reviews PLoS.pdf [file pone.0242927.s003.pdf]

Reviewer #1:

The writings of this paper needs substantial improvement before it can be considered for publication. The current writing is almost unreadable.

We are sorry that the reviewer had difficulties understanding the manuscript, and would be happy to have more details about which parts in particular were hard to read. In the revision, we rewrote several paragraphs to improve readability, e.g., we now clearly and concisely describe the four estimators based on modified outcomes in Section 2.1.

Reviewer #2:

In this paper the authors propose a framework to identify pairwise interactions with a particular target variant, using a penalized regression approach. The benefits of the framework is demonstrated by comparing to state-of-the-art techniques for epistasis detection on simulated and real data. Overall, the paper is nicely structured, but there are several problems to be addressed.

Major problems:

1) According to the paper, the target SNP may be identified from the literature, experiments, or top hits in previous genome-wide association studies. It seems that this screening is mainly based on the main effect of the SNPs on diseases. However, it is possible that one SNP has only interaction effect, and no marginal effect on the disease. Such SNPs will not be included in the analysis. If assuming only SNPs with main effect may have interaction effects, then the analysis should be done only among all the target SNPs.

We agree with the reviewer that it is plausible that one SNP has only high-order interaction effects and no marginal effects on the disease. If we select the target gene in our method based on marginal effect, as we suggest, then indeed we will miss all interactions between SNP without marginal effects. This being said, we would like to insist that this limitation is only a consequence of the choice of the target SNP, and not of the method. If one decides to investigate a target SNP with no strong marginal effect, then the method would still be able to detect interactions with other SNP, independently of their marginal effects.

We can use other measures of association to detect such SNPs and choose them as targets in our method. For example, measures such as distance correlation and mutual information evaluate statistical independence between two random variables. This covers not only main effects, but also, epistatic effects which make the epistatic pair of SNPs and the phenotype statistically dependent. We have edited our manuscript to clarify this point (1st paragraph of Section 2.1).

2) In the line 338 of page 16/30, “The labels are 1 for the SNPs interacting with the target A, and 0 otherwise”. I didn’t see any results with these labels. It is very interesting to see how the method performs for SNPs interacting or not with target A separately.

We used the SNP binary labels in all of our simulations to obtain the ROC and PR curves in Figure 1 (and the corresponding tables in Appendix B). To construct those curves, we used the binary labels as ground truth and the scores provided by a method as predictions. These curves allow us to visualize how good the different methods are at retrieving the SNPs that truly interact with target A. We have edited our manuscript to clarify this point (“Evaluation” in Section 3.1).

3) The simulated haplotypes/genotypes exhibit the same linkage disequilibrium structure as the original data. Can the authors show how the power or type I error for the proposed method changes with linkage coefficient between the target SNP and other SNPs, and compare them with the other baseline models. It is interesting to understand the impact of linkage structure on these methods.

We said that 'the simulated haplotypes/genotypes exhibit the same linkage disequilibrium structure as the original data' to explain that the simulated genotypes have the same linkage disequilibrium (LD) patterns as the reference genotypes. We refer the reviewer to the following webpage https://mathgen.stats.ox.ac.uk/genetics_software/hapgen/examples/examples.html for a thorough comparison of the LD patterns between the two types of genotypes.

In all of our simulations, we filtered all SNPs within a three-cluster window of the target. The goal is to avoid any spurious associations owed to LD. We opted for this filtering scheme because it takes into account the LD structure and not simply the distance between two SNPs. Moreover, the eight causal SNPs in each scenario are uniformly sampled from the rest of the chromosome which spans a large window $\approx$33Mb. This also helps eliminate potential LD effects on statistical power.

4) In the simulation setting in 2.1.1 on page 14/30, target A only has interaction effect with X_v SNPs, then such target A might not go through initial screening and be included in the analysis. It might be better to also include main effect of target A in the model.

This is highly dependent on the screening strategy (see our answer to question 1 above). In our use case, our strategy only relied on interaction effects for the selection of the target. However, it might be interesting to examine the impact of the inclusion of the main effect depending on its amplitude and the disease model configuration.

Minor problems:

1) For ROC and PR figures, there should be x and y labels.

The figures indeed have x and y labels.

Reviewer #3:

The authors followed the work by Tian et al. (2014), and proposed to detect epistasis in genome-wide association studies (GWAS). While the work by Tian et al. (2014) originally attacked the case with a binary treatment in a designed clinical trail, the epistasis detection in GWAS is a completely different story: (1) although authors claimed that the dominant effect of a genetic mutation can be coded with a binary factor, the additive effect of biallelic mutations (especially in a populational GWAS) cannot be coded with a binary factor as, as in

a usual case, the minor allele frequency is used and coded with three different values, 0,1, and 2; (2) Most GWAS are concerned with a majority of non-common genetic variants (so that minor allele frequency rates are far less than 0.5 as in RNA-seq data), so that it is difficult, if not impossible, to consider E[Y|A=1, X] if we define A=1 implying any mutation at a specific SNP. For the aforementioned issues, the proposed method has very limited utility in GWAS. Instead, I would like to suggest the authors may consider some experimental GWAS in animals (like rats), as some special designs present binary genetic variants.

We thank reviewer #3 for suggesting experimental GWAS in animals as a potential application of epiGWAS, given the direct binary nature of the variants.

We think that epiGWAS can be useful in the context of populational GWAS because it allows the separate study of dominant and recessive mechanisms: dominant if the values 0 and {1,2} are respectively mapped to 0 and 1, and recessive if {0,1} and 2 are respectively mapped to 0 and 1. Moreover, the binarization is a common procedure in GWAS, in particular for the study of epistasis. For instance, Prabhu and Pe'er (doi: 10.1101/gr.137885.112) and Llinares-López (doi: 10.1093/bioinformatics/bty1020) use binarized genotypes. Achlioptas et al. (doi: 10.1145/2020408.2020521) even use locality-sensitive hashing (LSH) to transform the original genotypes into binary vectors. We have expanded our manuscript to describe binarization in more details (See 2nd paragraph of Section 2.1).

In addition, GWAS in model animals studies present their own challenges, due to the use of a limited number of strains, inbreeding, and limited sample sizes https://www.ncbi.nlm.nih.gov/pmc/articles/PMC3625632/ .

Regarding the fact that GWAS is often concerned with rare variants, we agree that this makes the problem of epistasis detection *in general* very difficult. Still many people are interested in epistasis detection from GWAS study. In our method in particular, as pointed out by the reviewer, a difficulty with rare variants is that inverse propensity score weights may be very large. This is the reason why we consider four strategies to regularize the problem and compare them empirically. Our experimental results suggest that the resulting methods outperform state-of-the-art methods like GBOOST.

Another issue: Line 167-168: It is not clear how the equations system leads to the three approaches. What are the unknown variables in this system?

We agree that this part was not very clear in the original submission. In the revision, we completely rewrote Section 2.2 to present more clearly and concisely the four variants of the modified outcome method, removing unnecessary information like the equation system. We refer to the citation Lunceford and Davidian (2004) the reader interested to know more about how these estimators were derived in the context of average treatment effect with inverse propensity score weighting methods.

Reviewer #4:

The paper proposes an interesting idea of using propensity score to transform GWAS phenotypes for testing epistasis effects. A similar idea has been proposed in the area of personalized medicine. Several alternative transformations of Y are proposed whose

performances are compared with both simulated and real data. This is a nice written paper with a method that can be potentially very useful in practice. But I do have some comments and concerns that may hinder the application of the proposed method:

1. Since transformed Y is based on estimated propensity scores, it is not clear if type I error can be well controlled or not, in particular when the dimension of X is high. I would not trust any P-values provided in the paper unless simulated data suggests that this should not be a concern.

The high dimensionality of X can indeed be problematic for the robust detection of interactions between X and the target A. For this reason, our approach makes use of penalized regression to select a finite set of SNPs and stability selection to create a confidence measure for the SNP selection. In addition, we do not provide P-values but instead directly use the areas under the stability selection curves as score. The question of providing P-values is interesting but, as the reviewer mentions, would probably require specific simulations for calibration (as provided by our ROC and precision-recall curves in the simulations). Since the P-values for a given dataset would probably depend on many factors, we prefer not to provide P-values at all and instead provide a ranked list of predictions to prioritize further investigations of the top hits.

2. How should we choose X for real GWAS data analysis?

For real GWAS analysis, we can choose X as the rest of the genome (the whole genome except the target A) or a given set of SNPs. The SNP set may correspond to a genomic region of interest *e.g.* gene, promoter region, or alternatively, a pathway. We clarify this in the first paragraph of section 2.1.

3. There is a conditional independence assumption under the original propensity score approach which is highly likely to not hold for GWAS data, should we be concerned? What if X and A are correlated?

New response: We thank the reviewer for this remark. The conditional independence assumption is not needed in our work. It is needed in the causal inference literature under the potential outcome framework to justify that the expectation of the difference D1=E[Y(1)-Y(0)] between two potential outcomes Y(1) and Y(0) under two treatment conditions A=1 and A=0 can be expressed as the difference D2=E[Y|A=1] - E[Y|A=0] of the expectation of the observed outcome Y given the two treatment conditions. In our setting, we do not assume potential outcome variables, and instead directly express the interaction effect in terms of D2. Hence we do not need the conditional independence assumption to justify that D2 is equal to D1.

4. A needs to be binarized. How should we combine the (three) genotypes and make them into two groups?

Please see our answer to Reviewer #3 above.
