# Supplementary material for: Novel methods for epistasis detection in genome-wide association studies
Source: PLoS One. 2020 Nov 30;15(11):e0242927. doi: 10.1371/journal.pone.0242927 (PMC7703915; doi:10.1371/journal.pone.0242927)
Supplement: Attachment — Submitted filename: Response to Reviewers.pdf [file pone.0242927.s004.pdf]

Lotfi SLIM
Centre for Computational Biology, Mines ParisTech
Translational Sciences, SANOFI R&D
60 boulevard Saint-Michel
75006 Paris, France

Paris, October 20th 2020

Dear editor,

We sincerely thank you for managing the whole review process. We also thank the reviewer for examining our manuscript and providing interesting feedback.

We would like to transfer the exact statement we included in the acknowledgement section of our original manuscript to the funding section. We also apologize for not having clarified the commercial affiliations of some of the co-authors. The amended funding statement is the following:

This study makes use of data generated by the Wellcome Trust Case-Control 509 Consortium. A full list of the investigators who contributed to the generation of the 510 data is available from http://www.wtccc.org.uk. Funding for the project was provided by the 511 Wellcome Trust under award 076113, 085475 and 090355.

In the form of salaries, L. S. and C. C. were supported in their research by SANOFI and JP. V. by Google. These companies did not have any additional roles in the study design, data collection and analysis, decision to publish, or preparation of the manuscript. The specific roles of these authors are articulated in the 'author contributions' section.

Additionally, as you suggested, our competing interests statement can be updated as follows:

The commercial affiliations of some of the co-authors do not alter our full adherence to all PLOS ONE policies on sharing data and materials.

The rest of our manuscript now meets the journal's style guidelines. We also updated the author affiliations.

The remark of the reviewer absolutely explains the gap in performance between the modified outcome (MO) and the outcome weighted learning (OWL) approaches. In OWL, the term d^* has the same sign as the term δ in MO. The sign commonality is not sufficient to filter out the common effects term $\mu(X)$. Despite this limitation, the OWL approach remains statistically valid, in the sense that its support recovery performance increases with the sample size (see supplementary material). In practical settings, we

naturally recommend the use of the MO approaches which linearity allow a complete filtering of the term $\mu(X)$, and consequently yield a better performance. We included this remark to our discussion section.

We remain at our disposal for any further enquiries.

Sincerely,

Lotfi Slim on behalf of all co-authors
